# Congenital Dermal Melanocytosis Exhibited in Two Patients with Hurler Syndrome: Clinical Characterization and Report of a Recurrent *IDUA* Allele in Colombia

**DOI:** 10.3390/ijms262110418

**Published:** 2025-10-27

**Authors:** Sara Vanegas, Diana Ramírez-Montaño, Alejandro Padilla-Guzmán, Harry Pachajoa

**Affiliations:** 1 Greenskin Centro Dermatológico, Cali 760032, Colombia; sarivanegas@gmail.com; 2 Departamento de Morfología, Universidad del Valle, Cali 760032, Colombia; diafel1520@hotmail.com; 3 Unidad de Medicina Genómica y Genética, Clínica Imbanaco, Cali 760042, Colombia; 4 Health Sciences Faculty, Universidad Icesi, Cali 760031, Colombia; alejandro.padilla@fvl.org.co; 5 Congenital Abnormalities and Rare Diseases Research Center (CIACER), Universidad Icesi, Cali 760031, Colombia; 6 Medical Genetics Service, Fundación Valle del Lili, Cali 760032, Colombia

**Keywords:** alpha-L-iduronidase, Mongolian spot, Hurler syndrome, Mucopolysaccharidoses

## Abstract

The potential association of congenital dermal melanocytosis as a marker for lysosomal storage disease in infancy is rarely studied. A few cases of congenital dermal melanocytosis in association with lysosomal storage diseases have been reported. GM1 gangliosidosis type 1 and Hurler syndrome are the most common underlying lysosomal disorders associated with dermal melanocytosis. We present two non-relative patients with Hurler’s Syndrome who exhibited cutaneous manifestations. Both cases had a recurrent genetic variant c.1045G>T (p.Asp349Tyr) in the *IDUA* gene, located in a highly conserved amino acid position. We encourage the role of cutaneous findings in early suspicion and detection of inborn errors of metabolism, as well as differential diagnoses in a newborn with this finding.

## 1. Introduction

Congenital dermal melanocytosis, or Mongolian spots (MSs), are benign, blue-gray skin macules appearing at birth that disappear as the child grows. They occur most commonly in the sacral area, back, thighs, legs, and shoulders, and can be solitary or multiple. Although congenital dermal melanocytosis is traditionally considered a benign condition, it could be a sign of underlying lysosomal storage disease (LSD), including Hurler syndrome, Hunter syndrome, and GM_1_ gangliosidosis type 1 [1].

Mucopolysaccharidosis is part of a family of inherited diseases (inborn errors of metabolism, specifically LSD) characterized by deficiency of lysosomal enzymes catalyzing the degradation of glycosaminoglycans, leading to its storage in the cells and excessive excretion in urine. Mucopolysaccharidosis type I (MPS I) is a recessive inherited disorder due to mutations in the *IDUA* (alpha-L-iduronidase) gene located in chromosome 4p16.3 [2]. These mutations lead to a deficiency in the enzyme alpha-L-iduronidase, which is required to cleave the mucopolysaccharide heparan sulfate and dermatan sulfate to avoid its accumulation in tissues [3]. It has been proposed [4] that accumulated heparan sulfate binds directly through glycosylation, tightly and specifically to receptors with tyrosin kinase activity as Trk, affecting signaling transduction and disrupting the paravertebral-dorsolateral-ventral and transdermal migration of melanoblasts (which starts at 2.5 weeks of gestation and is stimulated through chemotropic signals by peptide growth factors (such as the nerve growth factor, NGF) from the neural crest- and nerve-associated Schwann Cell precursors to the developing epidermis (melanocytes arrival at 8-10 weeks of gestation to dermis and at 10-14 weeks of gestation at epidermis; after the 20th week, melanocytes are not found in dermis due to the migration to the epidermis and their clearance by macrophages), arresting those to the dermis and resulting in extensive and persistent lesions (dermal melanocytosis) [5,6,7]. Also, this results in an abnormal increase in NGF activity leading to the development of large neural processes related with the onset, severity, and disease course of lysosomal storage diseases [5,6,7].

MPS I-Hurler syndrome is the most severe spectrum of this metabolic genetic disease, with an incidence of 1/100,000. The disease has progressive involvement, resulting in rapid deterioration; but at birth, the patient’s appearance is normal. The early characteristics can manifest in the first few months of life, including hepatosplenomegaly, joint movement limitations, intellectual disability, deafness, skeletal deformities, hernias, coarse facial features, and cornea clouding [8]. Typically, infants in their first year of life present with a progressive decline in cognitive and motor skills, recurrent respiratory infection, and cardiac disease [9].

We present two cases, including a five-year-old boy and a two-year-old girl, of dermal melanocytosis, which led to early diagnosis of Hurler’s syndrome. Cardiovascular and neurological compromise was noted in both cases; biochemical confirmation was performed, and gene sequencing identified a recurrent variant in the *IDUA* gene in both patients.

## 2. Case Presentation

### 2.1. Case 1

The proband is an Afro-American 5-year-old boy, born at 37 weeks as the second child of third-degree consanguineous parents from Barbacoas, Nariño, Colombia (a town located on the Pacific Coast with a total population of 38,708 inhabitants). The mother and father were 32 and 33 years old, respectively. The mother, otherwise healthy, had a history of gestational diabetes and preeclampsia but the rest of the pregnancy and cesarean delivery were uncomplicated. His birth weight was 4.3 kg (97th centile) and his length was 57 cm (99th centile). Clinical findings at birth included coarse face, umbilical and inguinal hernia, and widespread MS extending over the back and the upper and lower extremities. The patient exhibited recurrent respiratory infections. These infections started at 6 months of age, and included two episodes of bronchiolitis, and one episode of pneumonia.

**Figure 1:**
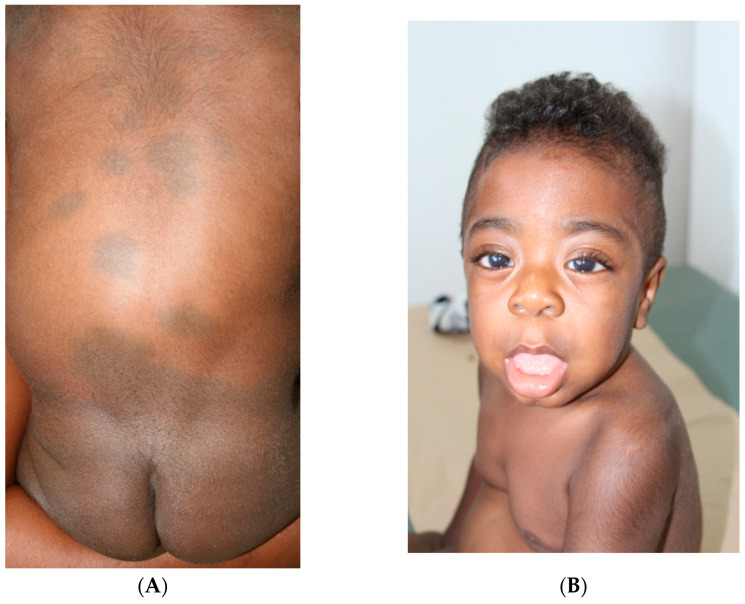
(**A**). Image showing patchy Mongolian spots involving the back and gluteal region in patient 1. Note the associated dorsal kyphosis. (**B**). Coarse facial features of patient 1: mild corneal clouding, prominent eyes with hypertelorism, depressed nasal bridge, and thickened tongue and lips.

At one year of age, he was medically assessed. His weight was 13 kg (48th centile) and his length was 80.5 cm (27th centile). The physical examination revealed generalized MS mainly located on the limbs and back region (see Figure 1A). Other findings included coarse facial features, macroglossia, broad nose and lips, smooth philtrum, corneal opacity, pectus excavatum, rales on lung bases, reducible umbilical and left inguinal hernia, and testicular bilateral hydrocele (see Figure 1B). Osteomuscular examination revealed kyphosis, right fifth finger clinodactyly, and articular stiffness in all limbs.

At 13 months of age, urine electrophoresis analysis for mucopolysaccharides identified two single bands visualized with Alcian blue and migration; these bands were chondroitin sulfate and dermatan sulfate/heparan sulfate. An enzyme assay established the definitive diagnosis of MPS I. New generation sequencing (NGS) of the *IDUA* gene was performed in which a pathogenic missense homozygous variant in the *IDUA* gene was identified, c.1045G>T (p.Asp349Tyr). This variant was confirmed by Sanger sequencing using an ABI 3500 sequencer (Applied Biosystems, Thermo Fisher Scientific, Waltham, MA, USA). Bioinformatic analysis of the variant was performed: FATHMM classified the variant as “damaging,” while MutationTaster classified it as “causing disease”. Currently, it is classified as “pathogenic” according to the guidelines by American College of Medical Genetics and Genomics (ACMG) (PS3, PM1, PM2, PM3, PM5, PP2, PP3, PP5) [10]. Enzyme replacement therapy (ERT) started at this age, improving respiratory symptoms and motor function. At 15 months, hematopoietic stem-cell transplantation (HSCT) was performed but unfortunately, 1 month later, graft failure was developed. At five years old, the patient underwent a genetic consultation. The physical examination showed similar features reported at the first consultation. His height was 111 cm (36th centile) and his weight was 21.9 kg (75th centile).

### 2.2. Case 2

The proband is an Afro-American, two-year-old girl born from the first pregnancy of a 29-year-old mother and a nonconsanguineous 24-year-old father from Barbacoas, Nariño, Colombia. Vaginal delivery at the 37th week of gestation went without complications, and the anthropometric parameters at birth were a weight of 3.595 kg (25th centile) and a length of 47 cm (25th centile). The perinatal period was uncomplicated. Clinical findings at birth included a coarse face, a giant umbilical hernia, a widespread MS extending over the back region, and hyperpigmented patches in the lower extremities. The patient exhibited recurrent respiratory infections which started at 1 month of age and included two episodes of bronchiolitis with a negative Asthma Predictive Index.

**Figure 2:**
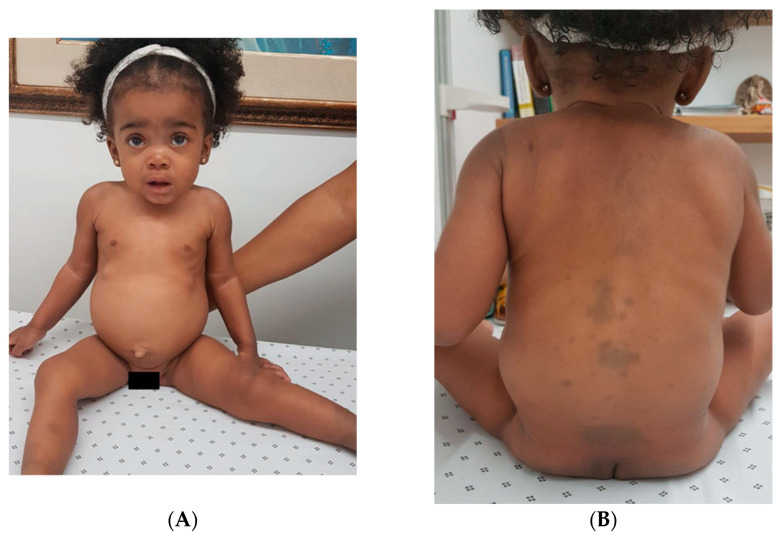
(**A**) Facial features of the two-year-old proband, frontal view, showing coarse facial features, broad forehead, synophrys, hypertelorism, broad and depressed nasal bridge, posteriorly rotated ears, and prominent abdomen with an umbilical hernia. Mongolian spots are observed on the inferior extremities. (**B**). Blue cutaneous patches extend over the posterior trunk, especially on the thoracolumbar area and buttocks.

She was referred from the Pediatric Cardiology Department to the Genetics Department for the clinical history of congenital mitral insufficiency, small patent ductus arteriosus, and ostium secundum atrial septal defect, associated with inguinal hernia and congenital talipes equinovarus. At 6 months of age, she was evaluated by a geneticist. The physical examination revealed coarse facial features, low-set ears with overfolded helix, protruding tongue, broad nose and lips, smooth philtrum, corneal opacity, giant umbilical hernia with a ring of 15 cm in diameter, right inguinal hernia, kyphosis, and an extensive MS in the back region were noted (See Figure 2A,B), and hyperpigmented patches in the lower extremities.

At one year of age, a deficiency in alpha-L-iduronidase enzyme activity in leukocytes was identified, and the diagnosis of MPS I was confirmed. NGS of the *IDUA* gene was performed and revealed a pathogenic missense homozygous variant in the gene, c.1045G>T (p.Asp349Tyr). This variant was confirmed by Sanger sequencing with an ABI 3500 sequencer (Applied Biosystems, Thermo Fisher Scientific, Waltham, MA, USA). Bioinformatic analysis of the variant was performed; FATHMM classified the variant as “damaging,” while MutationTaster classified it as “causing disease”. Currently, it is classified as “pathogenic” according to the guidelines by the American College of Medical Genetics and Genomics (ACMG) (PS3, PM1, PM2, PM3, PM5, PP2, PP3, PP5) [10]. ERT started at 13 months old, resulting in improved respiratory symptoms. The patient was considered for HSCT to improve neurological compromise and was surgery performed at two years old without complications. At 30 months old, the patient underwent a genetic consultation. Her height was 82.4 cm (1st centile) and her weight 12.2 kg (27th centile).

Both cases are currently under surveillance by a multidisciplinary health team, including specialists in the fields of pediatric pulmonology, hematology/oncology, cardiology, dermatology, and medical genetics.

## 3. Discussion

MS, or Mongolian spots, or dermal melanocytosis, are nonblanching grayish-blue macules with irregular margins that present in some newborns at birth or during the first few weeks of life. These macular lesions most commonly appear in the lumbosacral and gluteal area, but can also be found on the posterior thighs, abdomen, arms, legs, back, and shoulders; regression happens in early childhood (rarely persists up to 6 years) [7,11,12,13]. MS are more frequently observed in Japanese, African American, and Hispanic infants than in Caucasian neonates-infants [5,7]. Several studies have shown that there can be an association between certain birthmark types and some underlying genetic conditions [14]

Recent research data have shown that extensive, persistent, and atypical dermal melanocytosis (instead of a gluteal or lumbosacral, a dorsal-ventral localization in the abdomen, shoulders, arms, legs, back, and face; indistinct borders and persistent or progressive behavior) can be associated with LSD, most commonly, MPS I and GM1 gangliosidosis type 1 [3,4,6,7,9,15,16,17]. These atypical localization of MS were presented in our cases. Ashrafi et al. have reported a case of a five-month-old male infant with the typical features of MPS I, with extensive MS on his back, buttocks, and distal extremities [15]. Numerous similar cases have been reported with a similar phenotype that was found in our cases [4,9,17,18]. Herein, we report two cases of Hurler Syndrome with a recurrent homozygous variant in the *IDUA* gene, c.1045G>T. Both patients presented extensive MS on the sacrum, which suggested at birth an early possible association with LSD (see Figure 1A and Figure 2B). Dermatologic manifestations in patients with MPS I can present as pebbling of the skin, ivory-colored papules, acrosclerotic changes over the hands, and persistent dermal melanocytosis [7].

Other inborn errors of metabolism that present with this feature are Niemann–Pick disease [19], alpha-Mannosidosis [20], mucolipidosis II [21], and other forms of MPS like type 2 (Hunter syndrome) and type 4 [22]. Table 1 summarizes the clinical conditions that present with congenital dermal melanocytosis, including other dermatological diseases like phacomatosis [23,24] and congenital nevi, including Spitz nevus [25], Becker nevus [26], congenital melanocytic nevus [27], and other melanocytic lesions like *café-au-lait* spots [28], hematomas, and congenital hemangiomas [29,30]. All these entities are part of differential diagnosis and reflect the clinical heterogeneity and etiology of this finding.

Hypotheses suggest that the enzyme deficiencies in these newborns and their accumulated products have a negative effect on melanocyte migration during embryological development [4]. Hanson et al. have postulated that NGF might play a significant role in melanocyte migration, given that they have been shown to possess receptors for this neuropeptide [5,6,7]. They hypothesized that the accumulated metabolites in MPS 1 and GM1 gangliosidosis type 1 bind tightly to Trk, which also serves as a molecular receptor for NGF. This binding leads to an unusual increase in NGF activity and plays a significant role in disrupting melanocyte migration during embryogenesis [4,6,7]. Although several mechanisms in skin homeostasis through the neuropeptide axis have been proposed [31,32], this is the mechanism most studied for congenital dermal melanocytosis in MPS-I [5,6,7]; yet, it is a process that is poorly understood due to the limited data about this disease, as in other innate errors of metabolism.

Most newborns with MPS I are asymptomatic at birth so it should be suspected in children with atypical cutaneous findings related to anomalous melanocyte migration, skeletal deformities, coarse facial deformities, and corneal opacity. Nevertheless, onset characteristics could vary, and diagnosis may be difficult. Biochemical laboratory testing should be the first screening test performed and radiographical evaluation for skeletal dysplasia can also be performed. Genetic analysis screening of the *IDUA* gene is cost-effective for an early diagnosis which is extremely important to the progression of the disease by allowing prompt and adequate treatment.

The variant (c.1045G>T) identified in the two Afro-American patients of our cases is a very infrequent variant reported previously [33]. Therefore, its identification in two patients from the same geographic area could be explained by the low population, isolated geographical area, and armed conflict in this zone, along with the suggestion of a founder effect.

In conclusion, an unusual presentation of dermal melanocytosis is often the clue to an underlying lysosomal systemic disorder such as Hurler disease. In LSD, MS are more extensive, deeper blue, and last for many years compared with benign MS [4,22]. Early recognition of lysosomal storage disease based on MPS I phenotype and dermatologic hallmark sign should help for an early accurate diagnosis. Although the prevalence of MPS I is low, our cases suggest a founder effect in the Colombian Pacific Coast.

## Figures and Tables

**Table 1:** Clinical conditions that can present congenital dermal melanocytosis.

Disease	Etiology/Gene	Inheritance	Clinical (Not Dermatologic) Features	Dermatologic Features	Ref.
**Inborn Errors of Metabolism**					
**GM1 gangliosidosis (Type 1 and 2)**	Pathogenic variants in *GLB1* gene.	AR	Coarse facial features, gingival hypertrophy, corneal clouding, cherry-red macula, hepatosplenomegaly, vacuolated lymphocytes, and skeletal dysostosis in addition to a history of psychomotor regression.	Classical congenital dermal melanocytosis in the back, and gluteal region, and extremities. It presents with one or more macules with round, oval or angled morphology. Variable size from 1 to 20 cm with poorly defined edges (largest are better delimited). Homogeneous gray-blue coloration that is not accentuated on Wood’s lamp examination. The macules are present at birth and usually disappear at the age of 3 to 4 years.	[4]
**MPS 1**	Pathogenic variants in *IDUA* gene.	AR	Coarse facial features (including macrocephaly with bulging frontal bones, depressed nasal bridge with broad nose with flared nostrils, thick lips), corneal clouding, skeletal dysostosis, thoracic-lumbar kyphosis, short stature, hepatosplenomegaly, hernias, cardiomyopathy and valve abnormalities, sensorineural hearing loss, enlarged tonsils and adenoids, upper airway recurrent infections with increased secretions. Neurologic involvement: delayed psychomotor development usually obvious by the age of 12 to 24 months, language skills very limited, with progressive mental decline leading to a severe intellectual disability.		[4]
**MPS 2**	Pathogenic variants in *IDS* gene	XLR	Coarse facial features, recurrent upper airway infections with increased secretions, hearing loss, hepatosplenomegaly, cardiac involvement, decrease joint mobility and global delay of developmental milestones by the age of 2 years. Neurological features of MPSII are represented by marked behavioral disturbances, such as hyperactivity, obstinacy, and aggressiveness.		[23]
**Niemann-Pick disease (Infantile form)**	Pathogenic variants in *NPC1* or *NPC2* genes.	AR	The neonatal signs range from transient unexplained jaundice to severe cholestatic hepatopathy. Isolated hepatomegaly or splenomegaly in the infantile period associated with developmental delay in motor milestones and central hypotonia are the first neurologic symptoms. Also present are the loss of acquired motor skills, spasticity, intention tremor, and hearing loss. Brain imagery may show leukodystrophy. Frequently in late infantile period hepatosplenomegaly, ataxia, clumsiness, and frequent falling. Hearing loss, dysarthria with delayed speech and dysphagia are often present; focal or generalized seizures (sometimes fatal), cataplexy, and vertical supranuclear gaze palsy (VSGP) are usually present, while mental impairment and behavioral disturbances become more marked.		[20]
**Alpha-Mannosidosis.**	Pathogenic variants in *MAN2B1* gene	AR	Immunodeficiency, facial and skeletal abnormalities, hearing loss and intellectual disability.		[21]
**Mucolipidosis II (I-Cell disease)**	Pathogenic variants in *GNPTAB* gene	AR	Stunted growth, skeletal joint abnormalities, coarse facial features, corneal clouding, intellectual disability, hepatomegaly, cardiomegaly and respiratory infections, similar to MPS. Severe progressive neuropathy and oculoskeletal dysfunction are recurrent features.		[22]
**MPS 4**	Pathogenic variants in *GALNS* gene.	AR	Coarse facial features, short stature, skeletal dysplasia with short stature, spinal cord compression, pectus carinatum, kyphoscoliosis, genu valgum with joints are usually lax and very flexible (hypermobile). In severe phenotypes, airway compromise (Restrictive pattern), and later valvular heart disease are the leading causes of morbidity and mortality.		[23]
**Sjogren-Larsson syndrome.**	Pathogenic variants sin *ALDH3A2* gene.	AR	Congenital ichthyosis, delayed psychomotor development, due to spastic diplegia, seizures, moderate or severe intellectual disability.		[24,25]
**Phacomatosis**					
**Phacomatosis pigmento-vascularis**	Unknown	NA	Ocular dysfunction, usually bulbar melanosis, glaucoma, retinal hemangioma and hearing loss.	Disseminated vascular nevus (port wine stain type) associated with pigmentary nevi (epidermal nevus, nevus spilus, or dermal melanocytosis).	[24,25]
**Phacomatosis pigmento- pigmentaria**	Unknown	NA	Non-extracutaneous clinical features.	Two coexisting pigmentary nevi (epidermal nevus, nevus spilus, or dermal melanocytosis).	[24,25]
**Congenital hemangioma**	Unknown	NA	Visceral hemangiomas. As part of PHACE association: neurocutaneous condition with facial hemangiomas associated with a spectrum of posterior fossa malformations, arterial cerebrovascular anomalies, cardiovascular anomalies, and eye anomalies. Dysraphic myelodysplasias associated with urogenital and anorectal anomalies.	Most common benign tumor in childhood. It usually presents as a single flat, slightly rounded or oval lesion. Pink central area with less telangiectasias, and dark purple surrounding area with greater presence of telangiectasias. Whitish external area. Location mainly in the head and neck (43%) and extremities (38%).	[30,31]
**Melanocytic Nevus**					
**Blue Nevus (BN)**	Patogenic variants in *GNA11* or *GNAQ* gene.	NA	Non-extracutaneous clinical features.	The common BN is a well-demarcated, slightly raised, or dome-shaped circumscribed symmetric bluish or bluish-black papule, usually measuring less than 1 cm in diameter. Preferred anatomic locations include the dorsal aspects of the hand and feet, the face and the scalp. Women > men.	[28]
**Nevus of Ota/Nevus of Ito**	Unknown	NA	It can be associated with vascular malformations in Sturge Weber syndrome and Klippel Trenaunay syndrome.	Nevus of Ota Blue represents a unilateral, patchy gray or brown irregular dermal melanosis, often spotted skin discoloration located on the face in the distribution of the first and second branches of the trigeminal nerve, the sclera on the affected side might also have a bluish discoloration. Nevus of Ito differs from nevus of Ota in its distribution that is confined to the neck, shoulder and proximal arm region. Can be in isolation or together.	[28]
**Spitz Nevus**	Unknown	NA	Non-extracutaneous clinical features.	Solitary, rounded or oval papule, with a smooth surface. It may also be verrucous, with mild scaling, crusting, or erosion. Its color can vary from pink to reddish-brown or purple-red and its growth may be slow or fast It appears in the first two decades of life, although it may appear in adulthood in 1/3 of the cases. This is rare at birth.	[26]
**Becker Nevus Syndrome**	Hormone dependent disorder (androgens)	NA	Unilateral hypoplasia of breast or other cutaneous, muscular or skeletal defects, involve the same side of the body as the nevus, ipsilateral hypoplasia of the shoulder girdle or abscence of the pectoralis mayor muscle and ipsilateral hypoplasia of a limb, hemivertebrae or spina bifida oculta, fused or accesory cervical ribs, pectus excavatum, pectus carinatum, internal tibial torsion, and scoliosis.	Hyperpigmented hairy macula lesion, with well-defined and irregular borders. It is located predominantly on the anterior trunk or on the scapular region, lesions appear around eight years of age and become more evident in puberty. Other cutaneous findings are granuloma annulare, basal cell carcinoma, malignant melanoma, lymphangioma osteoma cutis, and hypohidrosis.	[27]
**Congenital Melanocytic Nevus**	Pathogenic variants in *NRAS* or *HRAS* gene.	NA	High risk of melanoma, diffuse lipomatosis, hypertrophy of cranial bones, central nervous system malformations, such as arachnoid cysts, choroid plexus papilloma, cerebellar astrocytoma.	Brownish lesion with well-defined borders and hypertrichosis, the surface of the nevus may be papular, roughed, warty or cerebriform, most cases occur in the first two years of life. Its most frequent location is the torso, followed by the limbs and head, affects more than one body segment, some peculiar lesion led to the term in garment, described as bathing trunk, stole or coat sleeve.	[28]
***café au lait spots***	Pathogenic variants in *NF1*, *PTPN11*, *BLM* and *GNAS* gene.	AD	Neurofibromas and Lysch nodules are diagnostic hallmarks of neurofibromatosis. Short stature and cardiomyopathy could be seen in Noonan Syndrome. Precocious puberty and fibrous dysplasia should suggest McCune–Albright syndrome.	Flat hyperpigmented spots or macules, light brown in color, irregularly shaped skin lesions can occur anywhere on the body but appear most frequently on the trunk and extremities. Some have smooth, well-defined borders and others have more jagged edges. They may be present at birth but may also grow in number and size over time, most are benign.	[29]

AR: Autosomal recessive. AD: Autosomal dominant. XLR: X-linked recessive. NA: not applicable. MPS: Mucopolysaccharidoses.

## Data Availability

The original contributions presented in this study are included in the article. Further inquiries can be directed to the corresponding author.
